# Characteristics of incidentally found thyroid nodules in computed tomography: comparison with thyroid scintigraphy

**DOI:** 10.1186/s12880-017-0178-8

**Published:** 2017-01-21

**Authors:** Shahin Zandieh, Dina Muin, Reinhard Bernt, Karl Hittmair, Joerg Haller, Klaus Hergan

**Affiliations:** 1 Institute of Radiology and Nuclear Medicine, Hanusch Hospital Teaching Hospital of Medical University of Vienna, Vienna, Austria; 2 Department of Radiology, Paracelsus Medical University of Salzburg, Salzburg, Austria

**Keywords:** Computed tomography, Thyroid scintigraphy, Thyroid nodule, Incidentaloma, Technetium thyroid uptake

## Abstract

**Background:**

In our daily experience, the differentiation between a cold and hot nodule is a very important factor for further clinical management of the patient.

In this study, we compared the characteristics of incidentally found thyroid nodules detected on computed tomography (CT) to thyroid scintigraphy (TS).

**Methods:**

Diagnostic reports from chest CT with intravenous contrast and TS examinations performed from January 2013 to January 2016 were analyzed retrospectively. We identified 70 subjects: 50 with thyroid nodules and 20 control subjects without thyroid nodules. The examination time of the TS was a minimum of two to four months after a chest CT. Chest CTs were performed in the arterial phase after the application of contrast media.

**Results:**

Patients with a cold nodule had a significantly lower Hounsfield Unit (HU) Nodule(N)/Parenchyma (P) ratio values than the patients with a hot or warm nodule (*P* < 0.05). The cut-off HU N/P ratio value with the highest sum of sensitivity and specificity for the prediction of a functioning nodule was 69 (95% CI: 0.79–0.95).

**Conclusions:**

Our results imply that the HU N/P ratio of the thyroid nodule on the chest CT should be taken into account to assess the functionality of the nodule. A lower HU N/P ratio should alert the radiologist or nuclear medicine physician to the possibility that the nodule might be cold and thus more prone to malignancy.

## Background

Thyroid scintigraphy (TS) is a nuclear medicine method that generates a functional depiction of a thyroid based on the uptake degree of different radionuclides. TS as a diagnostic procedure helps the physician obtain information about the thyroid’s functional state. In addition, it determines the size, shape, and position of the thyroid gland. TS can define the degree of function in a thyroid nodule that is palpable or found incidentally during a non-nuclear imaging procedure. Hot nodules are more often benign than cold lesions are. The most commonly used substance is radioactive technetium-99 m [1–4]. After accumulation in the thyroid gland, gamma radiation is recorded by the gamma camera so that the thyroid is presented as an image and the function of the thyroid may be assessed. Simultaneously, the amount of radionuclide is measured in the thyroid gland, and the technetium thyroid uptake (TcTU) is determined.

The thyroid nodule is an anomalous growth of thyroid cells. Most of the thyroid nodules are benign; a small portion of thyroid nodules could inclose malignant cells. Most thyroid nodules cause no noticeable symptoms. Nodules in the thyroid are mostly found incidentally during routine physical examinations or during a diagnostic modality such as computed tomography (CT) or sonography, which have to be done for entirely unrelated reasons. Thyroid nodules could cause hyperthyroidism due to increased production of thyroid hormones. However, most thyroid nodules are actually non-functioning. Thyroid nodules are an extremely common thyroid disorder. The frequency of these incidentalomas has ranged from as low as 2% to as high as 67% [5].

Thyroid incidentaloma is a non-symptomatic lesion that is detected in a diagnostic modality while evaluating for non-thyroid-related issues. In the majority of cases, they are found during ultrasound examinations of the neck, followed by CT, magnetic resonance imaging (MRI) and 18 F-Fluor-Desoxyglucose positron emission tomography (18 F-FDG-PET).

A couple of studies have attempted to evaluate the diagnostic value of CT to differentiate malignant thyroid nodule lesions from benign thyroid nodules. In these studies, the histopathological results have been used as a reference standard [5]. Davis et al. reported a low possibility of malignancy in these thyroid nodules depending on other risk factors in their study. The histopathological results of most of these malignancies were small papillary carcinomas [6].

Knowing the type of nodule (i.e., cold, hot, or warm) is critical in the clinical routine. About 13% of cold nodules have the risk of malignancy [7]. In addition, identifying the characteristics of the nodule type is also important for further thyroid nodule management. Although the presence of a cold nodule could be due to benign conditions, such as cysts, macrocalcification, and less commonly, areas of fibrosis or changes after thyroiditis, a malign lesion cannot be excluded. A fine-needle biopsy is often necessary.

The prevalence of incidentally found nodules in the thyroid has been reported in a couple of mainly CT- and MRI-based studies. Yoon et al. [8] found incidental nodules in the thyroid nodules in 16% of participants in their study. They examined 734 patients who were not known to have thyroid disease using a 16-slice CT scan. They reported that 9% of the incidentally found thyroid nodules were malignant. 123 CT scans and 108 MRI examinations of the neck were used in the study by Youserm et al. A prevalence of 16% for incidental thyroid nodules was reported by this study group [9].

In our daily experience, the differentiation between a cold and hot nodule is a very important factor for further clinical management of the patient. In this study, we compared the characteristics of incidental thyroid nodules detected on CT to TS. To our knowledge, this is the first study that conducts such a comparison.

## Methods

### Ethical issues

The local ethics committee granted ethical approval for this study. Informed consent was obtained from all participants. This study conformed to the Declaration of Helsinki.

### Patient selection

We evaluated 70 subjects. In our study, we searched retrospective chest CT examinations performed from January 2013 to January 2016 that contained the words “thyroid nodule” in the diagnostic report. In 50 reports where one thyroid nodule was diagnosed, the patients also had TS. We identified and included 50 patients (19 men, 31 women; age range: 51–90 years; mean age: 67 years) in our study. The mean body mass index (BMI) of the patients was 26, and the mean weight was 68 kg. At the time of the CT and TS examinations, all patients were unmedicated. No treatment was performed between the two examinations. In addition, we added 20 control subjects (16 men, 4 women; age range: 52–77 years; mean age: 64 years) without thyroid nodules in CT and Ultrasound examination. The mean BMI of the control subjects was 25, and the mean weight was 64 kg. Patients with cysts, parenchymal changes after surgery, and radiodine therapy have been excluded. We selected only patients with one nodule detected incidentally on chest CT examination.

### CT examination protocol

The CT scans were performed with a Brilliance 64-slice CT scanner (Philips Medical Systems Corporation, Cleveland, Ohio, USA). The following parameters for the 64-section multi-detector scanner were used: reconstructed slice thickness, 3 mm; reconstruction increment, 2 mm; x-ray tube current, 200 mAs; voltage, 120 kV; and collimation, 6.4 × 0.63 mm. A filtered back projection reconstruction technique was used.

The chest CT scans were done in the craniocaudal direction. The iodine contrast material dosage was 300 mgI/kg. The injection flow rate was 2.5 mL/s. The chest CT was performed during the arterial phase. Both arms were raised above the shoulder region. No artefacts were detected in the thyroid region on selected chest CT examinations. The average radiation dose associated with chest CT scanning was 7 mSV.

### Thyroid scintigraphy

The thyroid scintigraphy in our study was done with Tc-99-metastable pertechnetate. The scans were performed with a parallel high-resolution low-energy collimator at an energy setting of 140 KeV photo peak for Tc-99 m. The scan was done 20 min after the intravenous administration of 75–90 MBq Tc-99 m pertechnetate. The examination time of the TS was a minimum of two to four months after a chest CT. Nodules are depicted as zones of increased (hot), identical uptake compared to the uptake of normal paranodular thyroid tissue (warm) or decreased (cold) tracer uptake.

### Thyroid ultrasound

The US studies were performed with a Toshiba Aplio XG scanner (Toshiba Medical Systems, Tokyo, Japan) using an 18 MHz convex probe. The examinations of the patients were performed in the supine position. To locate the thyroid and to evaluate the thyroid nodule echogenicity, the real-time B-mode ultrasonography was used. The echogenicity of the nodule was reported as isoechoic, hypoechoic, or hyperechoic in relation to the thyroid parenchymal glands. The echotexture was described as homogeneous or heterogeneous. The US examination was performed on the same day as the thyroid scintigraphy.

### Image analysis

**Fig. 1:**
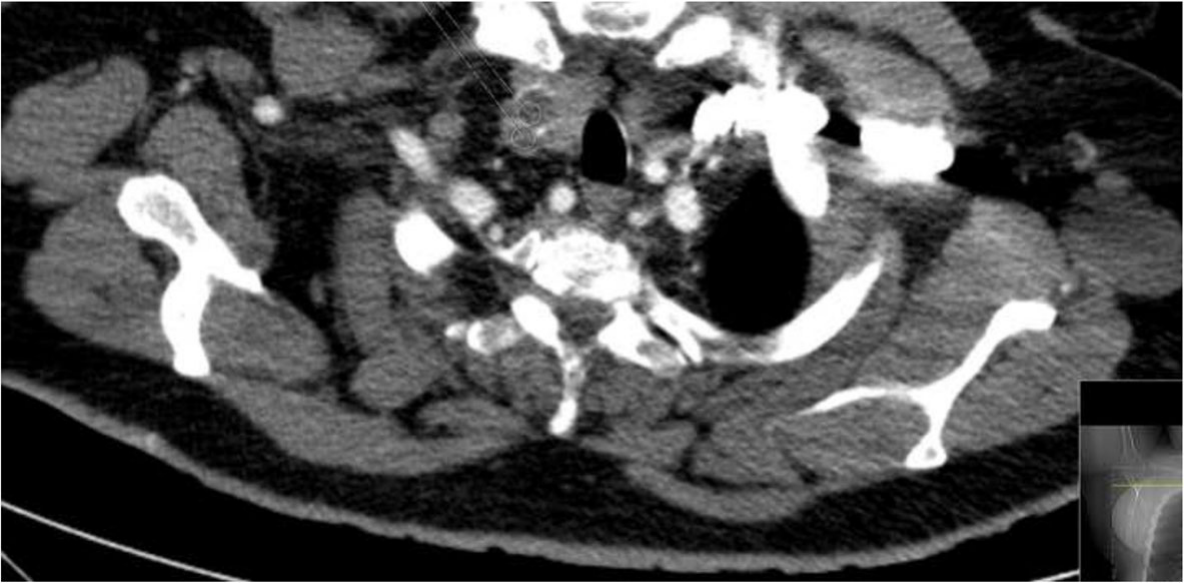
The CT image shows two ROIs in the thyroid nodule. Abbreviations: CT, computed tomography; ROI, region of interest

We have used our picture archiving and communication system (PACS) to fetch the CT images and transfer them to a standard viewer (Sectra; Version 15.2.4.1, Sweden). Two circular regions of interest (ROI) (5 mm) were placed in the center of the thyroid lesion, and another two ROI were placed in the thyroid normal parenchyma (5 mm) (Fig. 1). All chest CT studies were reviewed by a radiologist with 10 years of experience in chest imaging. We determined the HU density nodule to parenchyma ratio (HU N/P ratio) for two ROI in the thyroid nodule. We also determined the HU density nodule to control subject’s parenchyma ratio (HU N/P-control ratio) for two ROI in the thyroid nodule, and another two ROI were placed in the thyroid normal parenchyma.

### Statistical analysis

All data were recorded in a Microsoft Excel file, and the statistical analysis was conducted using SPSS (Version 22, IBM, USA) and ACOMED Statistic (Version 1, ACOMED, Germany) software. The results are presented as a box plot.

For quantitative analysis, we performed independent tests to estimate the differences in our results. To calculate the optimal cut-off value to maximize the sum of sensitivity and specificity and to estimate the area under the curve (AUC), receivers operating characteristic curve (ROC) analyses have been performed. Probability values of less than 0.05 were considered significant.

## Results

The study included 50 patients with chest CT and a thyroid incidentaloma.

The mean thyroid nodule size was 21 mm (min 11, max 50) on the CT of the chest in the axial plane. The hot nodules had a mean size of 22 mm, and the cold nodules had a mean size of 24 mm. The warm nodules had a mean size of 21 mm measured in the axial plane.

**Fig. 2:**
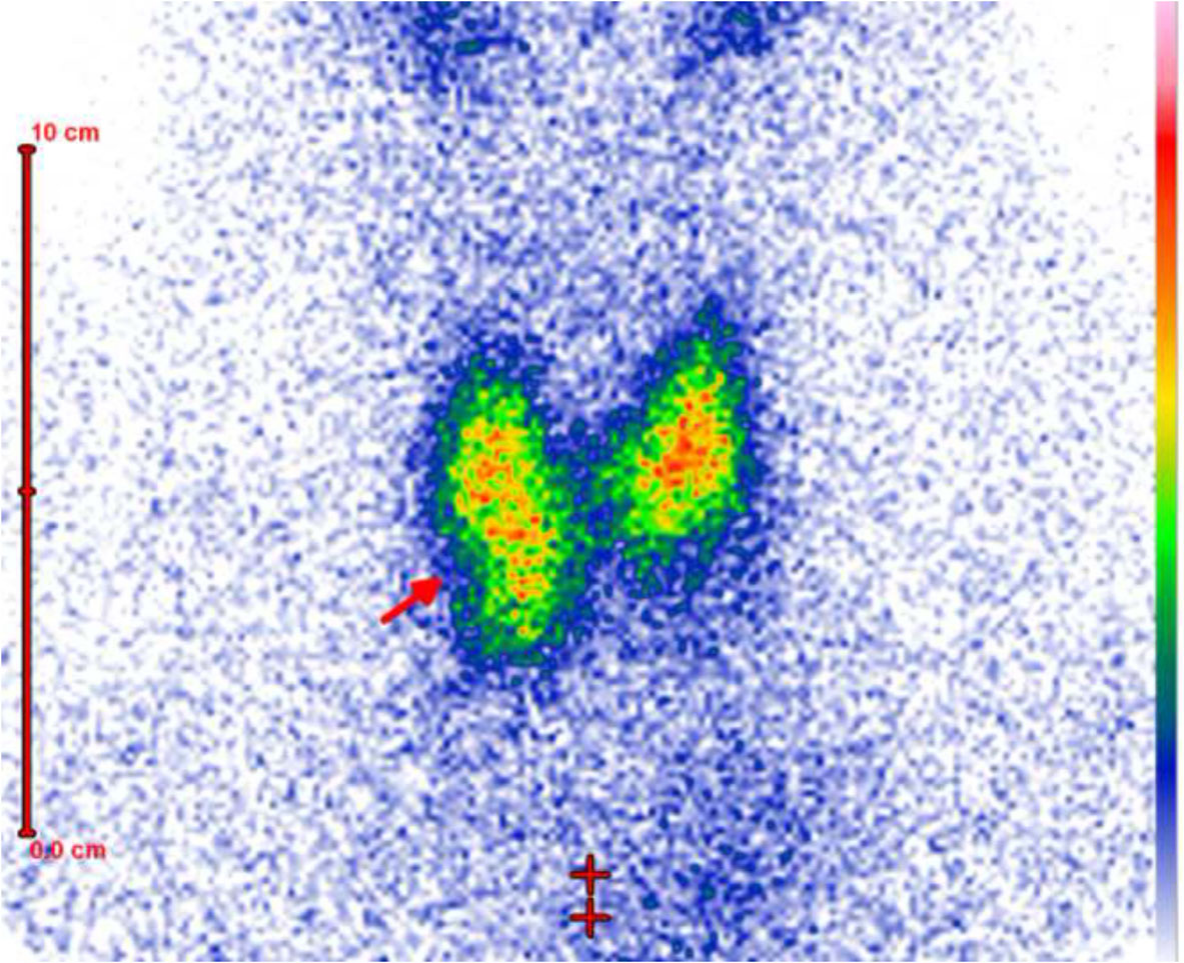
TS shows an area with diminished uptake in the right lobe of the thyroid laterocaudal which is consistent with a cold nodule. Abbreviations: TS, Thyroid scintigraphy

**Fig. 3:**
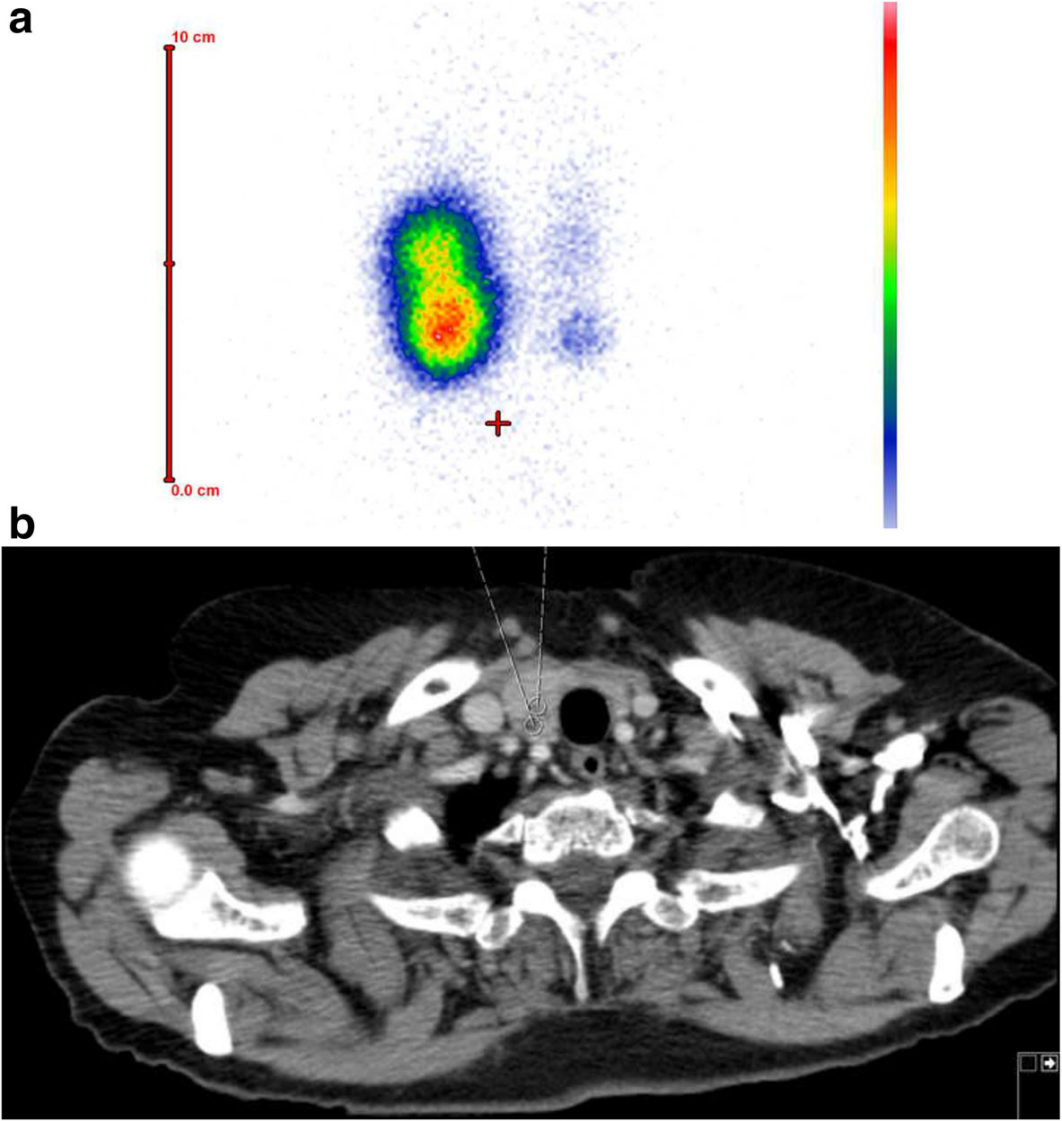
**a**, **b** TS shows an area with increased uptake in the right lobe of the thyroid caudal which is consistent with a hot nodule The chest CT depicts a nodule in the right lobe of the thyroid. Abbreviations: TS, Thyroid scintigraphy; CT, computed tomography

Hypofunctional nodules with diminished uptake (cold nodule) were seen in 20 patients (40%) (Fig. 2). Twenty-three patient (46%) showed a moderate uptake (warm nodule) on a Tc-99 m-Thyroid scan. Excessive uptake of Tc-99 m-pertechnetate was seen in 7 patients (14%) as defined by a hot nodule (Fig 3a, b).

On the ultrasound examination, the cold nodules were hypoechoic in 11 patients, isoechoic in 6 patients, and hyperechoic in 3 patients. The warm nodules were isoechoic in 12 patients, hypoechoic in 10 patients, and hyperechoic in 1 patient. Four of the patients with hot nodules were hypoechoic, and three were isoechoic.

The mean TSH value for patients with a cold nodule at the time of TS was 1.5 ± 1. Patients with a warm nodule had a mean TSH value of 2.2 ± 2 and with a hot nodule, a value of 0.8 ± 1.

Histopathalogy results of cold nodules from 16 patients revealed 2 patients with thyroid carcinoma, 1 with anaplastic carcinoma, and 1 with papillary carcinoma. In 2 cases inflammatory cells were present, and in 10 patients the results were compatible with non-malignant adenoma. Two patients with a cold nodule refused surgery and FNA. Only 4 patients with hot or warm nodules had biopsies indicative of non-malignant adenoma.

**Fig. 4:**
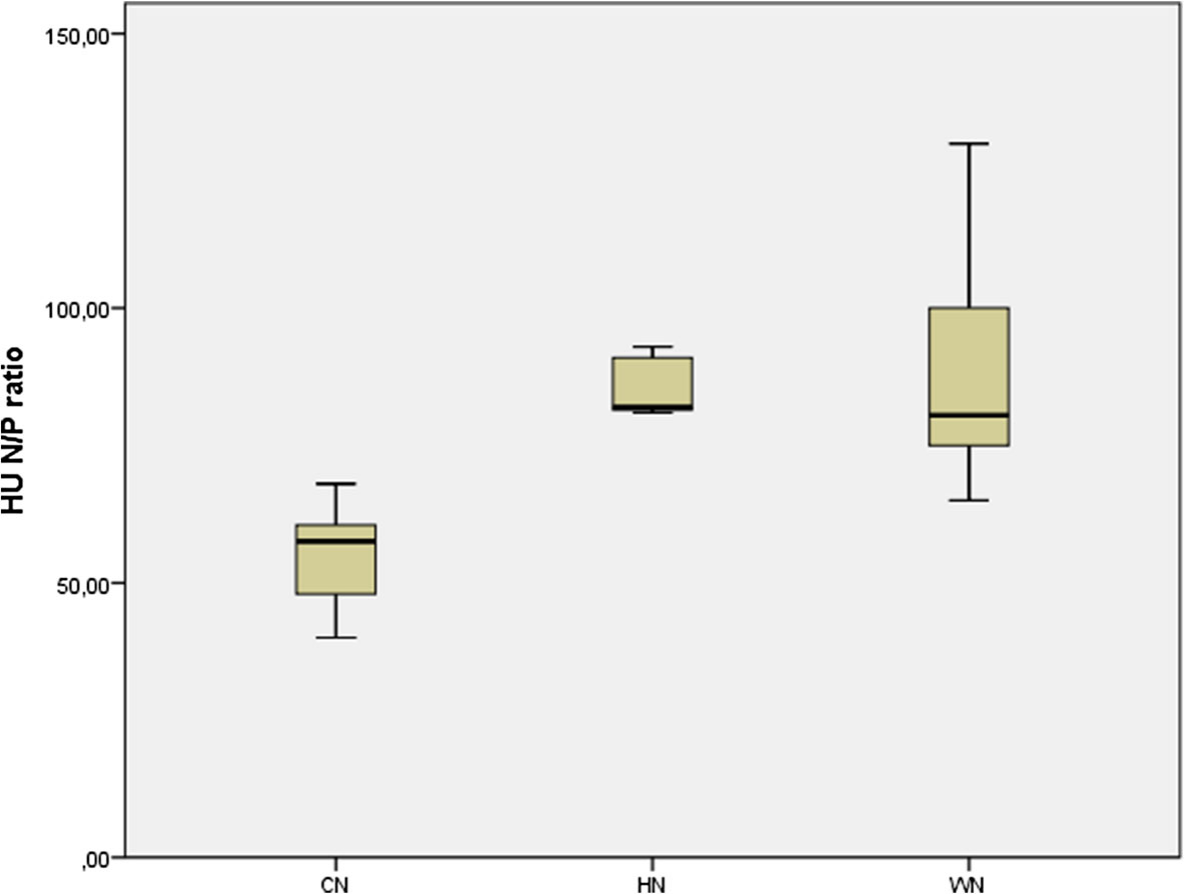
Boxplot graphics of HU N/P ratio values of CN, WN and HN. The HU N/P ratio values were lower in CN than to WN and HN (*P* < 0.05). Abbreviations: HU N/P ratio, Hounsfield Unit Nodule/Parenchyma ratio; CN, cold nodule; WN, warm nodule; HN, hot nodule

Cold nodules had a median 56 HU value on the chest CT. A median HU of 89 was detected for warm nodules on the chest CT. Hot nodules showed a median value of 86 HU on the chest CT examination. Cold nodules had a mean HU N/P ratio value of 56 ± 22 on the chest CT. A mean HU N/P ratio of 87 ± 20 was detected for warm nodules on the chest CT. Hot nodules showed a mean value of 86 ± 14 HU N/P ratio on the chest CT examination. The HU N/P ratio values were significantly lower in patients with cold nodules than the other groups (*P* < 0.05) (Table 1) (Fig. 4).

**Table 1:** Characteristics of CN, WN and HN detected incidentally on chest CT

	Patient	Mean size on CT	Isoechogenic	Hypoechogenic	Hyperechogenic	HU N/P ratio median
CN	20 (40%)	24 mm	6	11	3	56 ± 22
WN	23 (46%)	21 mm	12	10	1	87 ± 20
HN	7 (14%)	22 mm	3	4	0	86 ± 14

Abbreviations: *CN* cold nodule; *WN* warm nodule; *HN* hot nodule; *CT* computed tomography

Cold nodules had a mean HU N/P-control ratio value of 64 ± 25 on the chest CT. A mean HU N/P-control ratio of 94 ± 21 was detected for warm nodules on the chest CT. Hot nodules showed a mean value of 102 ± 16 HU N/P-control ratio on the chest CT examination. The HU N/P-control ratio values were significantly lower in patients with cold nodules than in the subjects of the other groups (*P* < 0.05).

**Fig. 5:**
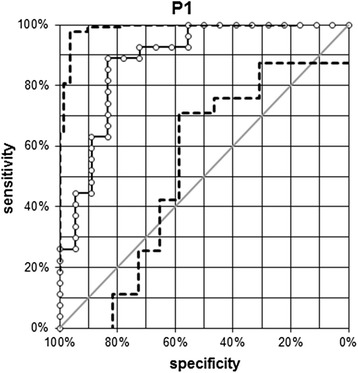
The ROC curve of the HU N/P ratio values assessed in patients with thyroid nodule. Abbreviations: ROC, Receiver Operating Characteristic; HU N/P ratio, Hounsfield Unit Nodule/Parenchyma ratio

The optimal cut-off HU N/P ratio value for which the sum of sensitivity and specificity was highest for the prediction of a functioning nodule (warm or hot) was 69 (95% CI: 0.79–0.95). For this cut-off value, HU N/P ratio value had a sensitivity of 89% and a specificity of 83%. To obtain a sensitivity > 90%, the best cut-off value was 65 (95% CI: 0.79–0.95) (94% sensitivity, 78% specificity) (Fig. 5) [10].

## Discussion

Thyroid incidentaloma is an unintentional finding that is not related to the original reason for performing a CT study. In the last decade, the evolution of diagnostic techniques has significantly improved diagnoses in endocrine thyroid disease.

The challenge facing the clinician is to avoid unnecessary diagnostic intervention, treatment, or both [11]. Frank et al. reported in a systematic review of 11 chest CT studies that had been performed for coronary artery disease and lung cancer screening had revealed that the proportion of patients with at least one incidental imaging abnormality requiring follow-up varied from 3% to 41.5% [12].

Thyroid abnormalities, such as nodules, cysts, calcifications, and diffuse glandular enlargement, are often detected on chest CT examinations. Thyroid nodules are found in approximately 16% of chest CT scans, occurring more commonly in women than men. Most incidentally found thyroid lesions are benign, with an approximately 9% to 11% prevalence of malignant lesions [12].

Once an incidental thyroid nodule is found, referral to a thyroid specialist is necessary. According to American Thyroid Association (ATA) guidelines, generally, only nodules >1 cm need further evaluation because they have a greater potential to represent clinically significant cancers compared with lesions <1 cm and because of cost/benefit considerations regarding the work-up of small nodules.

According to Austrian clinical guidelines, after detection of a thyroid nodule of over 1 cm, radionuclide imaging should be performed to characterize the thyroid nodule. If a nodule is cold, fine needle aspiration will be performed; otherwise, the nodule will be monitored on ultrasound besides there is a growth of the warm or hot nodule in the follow-up ultrasound examinations or FNA is recommended duo to the ATA guidelines. In radionuclide imaging, only 4% of hot nodules are known to contain malignant cells, compared with 16% of cold nodules [13].

Our results show no correlation between thyroid nodule functionality and US echogenicity. There was no correlation between the size of the thyroid nodule and functionality. The BMI values of the patient and the neck circumference measurements have no correlation to the thyroid nodule functionality.

Small amounts of free iodide are contained in an iodinated contrast medium. A 200-ml dose of a contrast medium containing 35 μg/ml provides 7,000 μg free iodide, equivalent to 45 times the recommended daily intake [14]. Our hypothesis was that iodine containing contrast media for chest CT accumulates in the thyroid due to the act of iodination, and this process could influence the HU values of the thyroid.

No CT feature is reliable in distinguishing cold from warm or hot thyroid lesions. In our study we tried to find a difference between the contrast uptakes of the thyroid nodule as detected incidentally by computed tomography and the degree of function of the same thyroid nodule on thyroid scintigraphy. Our data illustrate that there is a significant difference between the assessments, particularly, in the HU N/P ratio of the cold nodule to the hot or warm nodule. An HU N/P ratio values lower 69 should alert the radiologist of the presence of a cold nodule.

In addition, the results of the determination of the HU density nodule to control subject’s parenchyma ratio in our study were significantly lower in patients with cold nodules than in the subjects of the other groups.

Patients who have been referred to a thyroid specialist to diagnose the thyroid nodule could have had a prior chest CT for other reasons.

ATA and ETA (European Thyroid Association) guidelines suggest that nodules that are "hot or warm on scintigraphy" do not require a biopsy.

Our results suggest that the HU N/P ratio of the thyroid nodule on a chest CT should be taken into account to assess the functionality of the nodule before using thyroid scintigraphy. With further studies in the future, it may not be necessary to perform a TS in this context, which would avoid unnecessary radiation exposure and reduce costs.

In addition to the ultrasound characteristic of the nodule, a lower HU N/P ratio should alert thyroid specialists to the possibility that the nodule might be cold and thus more prone to malignancy, even using ATA and ETA guidelines for handling incidentalomas. In such cases, the biopsy could be done earlier.

In the study of Basharat et al., where a comparison was made between the results of TS and histopathology, the overall sensitivity on the TS was found to be 80%, specificity was 20%, positive predictive value was 10%, and negative predictive value was 90%. The overall accuracy was 26% [15]. In one study of TS, it was reported that the sensitivity was 100% and that the specificity was 24% [16].

False-positive TS is rare. Some artifacts and physiologic variants are known to consistently take up Tc-99-metastable pertechnetate, but these are easily discounted by experienced nuclear medicine physicians [17, 18].

Limitations of the study included a lack of pathological results and differentiation between benign and malignant thyroid nodules; however, these were not relevant to the aim of the study. Another limitation is calcification. This should not be captured in the ROI in order to avoid sustaining inadequate HU N/P ratio values. In our study, both arms were raised above the shoulder region. We have not included data about other arm positions. Therefore, further studies are necessary to determine the optimal cut-off HU N/P ratio for other arm positions.

We believe that with further studies, the guidelines could be reconsidered in the future.

## Conclusions

Our results imply that the HU N/P ratio of the thyroid nodule on the chest CT should be taken into account to assess the functionality of the nodule. A lower HU N/P ratio should alert the radiologist or nuclear medicine physician to the possibility that the nodule might be cold and thus more prone to malignancy, even using ATA and ETA guidelines for handling incidentalomas.

## Abbreviations

18 F-FDG-PET: 18 F-Fluor-Desoxyglucose positron emission tomography
ATA: American Thyroid Association
AUC: Area under the curve
CT: Computed tomography
ETA: European Thyroid Association
HU: Hounsfield Unit
MRI: Magnetic resonance imaging
PACS: Picture archiving and communication system
ROC: Receivers operating characteristic curve
ROI: Regions of interest
TcTU: Technetium thyroid uptake
TS: Thyroid scintigraphy
